# Kidney involvement during the course of febrile urinary tract infection

**DOI:** 10.1007/s00467-025-06695-4

**Published:** 2025-02-25

**Authors:** Gaia Pietropaolo, Anna Di Sessa, Paola Tirelli, Emanuele Miraglia del Giudice, Stefano Guarino, Pierluigi Marzuillo

**Affiliations:** Department of Woman, Child and of General and Specialized Surgery, Università Degli Studi Della Campania “Luigi Vanvitelli”, Via Luigi De Crecchio 2, 80138 Naples, Italy

**Keywords:** Urinary tract infection, Fever, Abdominal abscess, Pseudohypoaldosteronism, Acute kidney injury

## Abstract

Febrile urinary tract infections (fUTIs) are among the most common severe infections in the pediatric population, posing a significant risk to kidney health. The pathophysiology of fUTIs involves the direct bacterial invasion of kidney tissue, which triggers an inflammatory response. While this response is effective in eradicating the infection, it can also cause substantial kidney damage at multiple levels. In this review, we explore the various forms of kidney involvement in fUTIs, along with their clinical, radiological, and biochemical presentations. We also discuss the implications of these events for the long-term prognosis of kidney function. Understanding these manifestations can assist clinicians in making prompt diagnoses and initiating appropriate treatment to minimize complications such as sepsis, kidney scarring, and long-term kidney damage.

**Graphical Abstract:**

**Figure:**
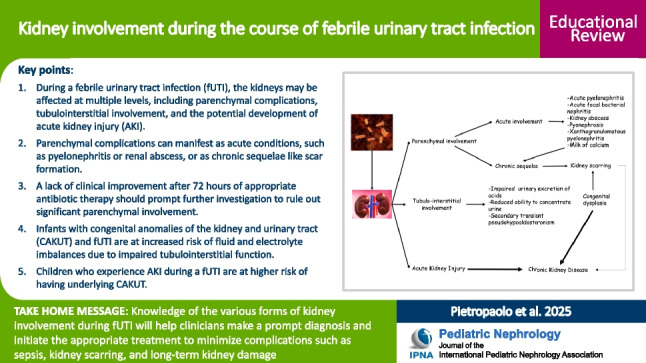
A higher resolution version of the Graphical abstract is available as Supplementary information

**Supplementary Information:**

The online version contains supplementary material available at 10.1007/s00467-025-06695-4.

## Introduction

When a febrile urinary tract infection (fUTI) occurs, the kidney parenchyma is typically affected, and patients may present with systemic signs such as fever and general malaise [1]. Prompt diagnosis and treatment are crucial to prevent complications, including sepsis, kidney scarring, and potentially long-term kidney damage [1, 2].

However, the impact of fUTI extends beyond the kidney cortex. The kidney medulla, including its distal and collecting tubules, may also be affected during fUTIs [3]. Furthermore, indirect effects such as the development of acute kidney injury (AKI) can impair kidney function [4].

This educational review aims to highlight and analyse the effects of fUTI on the kidneys, with the goal of refreshing clinicians’ understanding of the underlying pathophysiology. Such knowledge is essential for providing comprehensive care, which includes treating the infection, managing potential hydro-electrolyte imbalances, and preventing AKI. For instance, AKI prevention is pivotal, as it helps reducing the risk of chronic kidney disease (CKD) in this already vulnerable population [5]. This issue is particularly significant because congenital anomalies of the kidney and urinary tract (CAKUT) account for one-third of fUTI cases [1] and are frequently associated with congenital kidney dysplasia and CKD [1, 6–8].

## Parenchymal involvement

### Pathophysiology of the fUTI

#### Bacterial virulence and host susceptibility factors

**Fig. 1:**
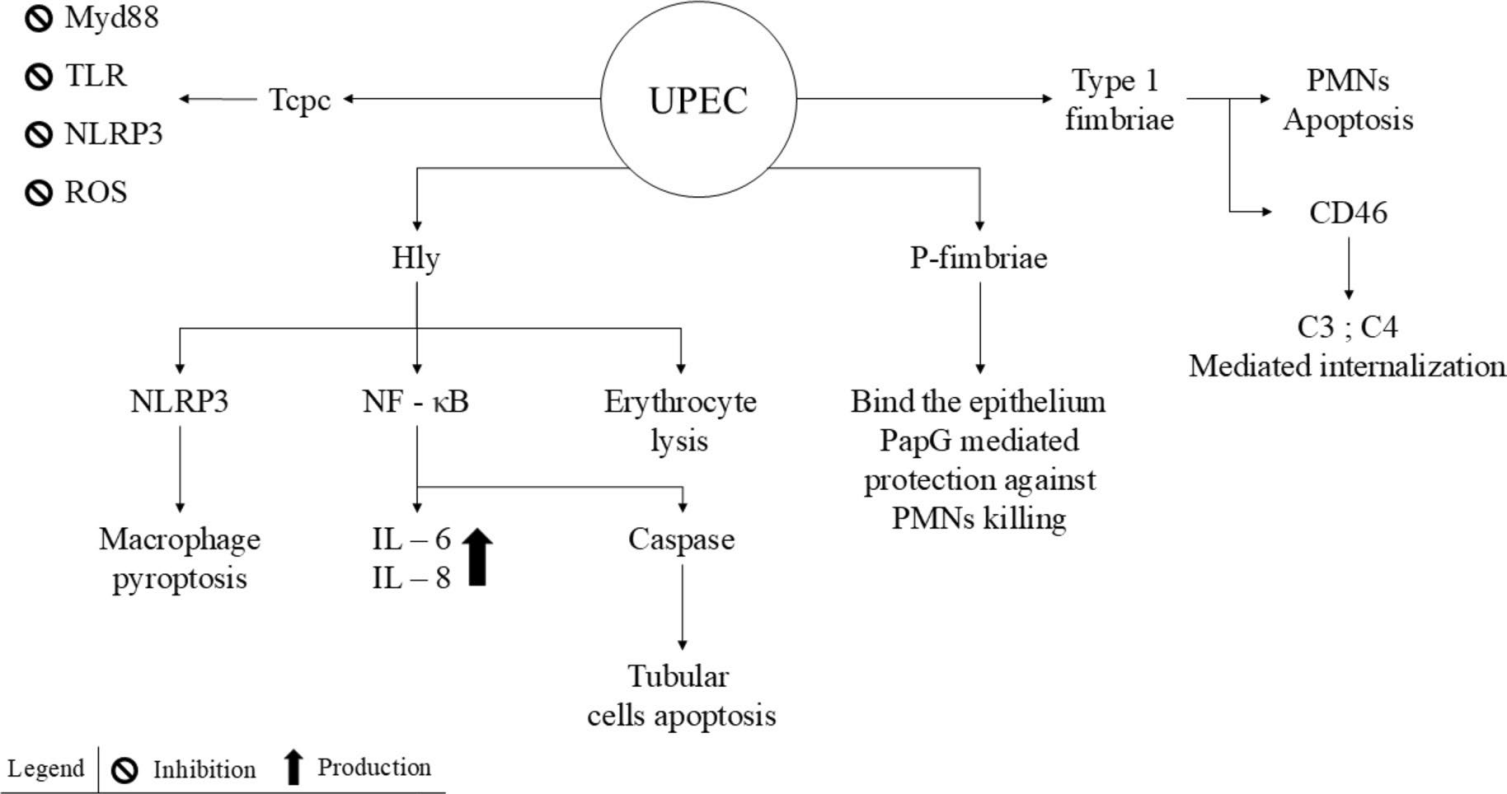
Bacterial virulence. *E. coli* invades the urinary tract using fimbriae as key virulence factor. Type 1 fimbriae facilitate the internalization of UPEC into the uroepithelium and induce apoptosis in PMNs. P fimbriae bind to the uroepithelium and enhance bacterial survival by protecting against PMN-mediated killing via the PapG protein. UPEC produces Hly, which exerts cytotoxic effects on erythrocytes, activates NF-κB to stimulate the production of pro-inflammatory cytokines, and triggers the NLRP3 inflammasome, leading to macrophage pyroptosis. Additionally, TcpC interferes with TLR signalling, a crucial pathway for initiating the pro-inflammatory response, by targeting MyD88. TcpC also inhibits the activation of the NLRP3 inflammasome and reduces the production of ROS and pro-inflammatory cytokines induced by LPS. α-hemolysin, Hly; interleukin, IL; myeloid differentiation factor 88, MyD88; NACHT leucin-rich repeat PYD protein 3, NLRP3; nuclear factor kappa-light-chain-enhancer of activated B cells, NF-κB; polymorphonuclear, PMN; reactive oxygen species, ROS; Toll/interleukin-1 receptor domain containing-protein C, TcpC; Toll-like receptor, TLR; uropathogenic Escherichia coli, UPEC

Febrile urinary tract infections are caused by uropathogenic *Escherichia coli* (UPEC) in approximately 80% of cases (Fig. 1) [9]. This strain of *E. coli* can migrate from the colon to the bladder, and may ascend through the ureters to the kidneys, causing acute pyelonephritis. The urinary tract’s primary defence mechanism is the hydrodynamic pressure generated by urine flow, which acts as a mechanical barrier between bacteria and the urinary epithelium [1, 10].

To overcome this barrier, bacteria must adhere to the tubular epithelium using surface ligands, primarily P fimbriae, type 1 fimbriae, and other adhesins. Certain *E. coli* strains express mannose-specific adhesins, recognized by phagocytic cells, which trigger the release of reactive oxygen species (ROS) and exacerbate tissue damage [11]. P fimbriae primarily mediate epithelial binding, while type 1 fimbriae enable interbacterial adhesion, which is essential for biofilm formation and colonization of perfused environments [12].

After adhering to the epithelium, UPEC invades the interstitium via complement-mediated internalization. This process is facilitated by type 1 fimbriae binding to CD46, which activates the production of C3 and C4. Type 1 fimbriae can also induce polymorphonuclear (PMN) cell apoptosis through a mannose- and lipopolysaccharide (LPS)-dependent pathway, enhancing UPEC virulence. Additionally, P fimbriae protect against PMN killing via the PapG tip adhesin, although the mechanism remains unclear [13].

One of UPEC’s primary virulence factors is α-hemolysin (Hly), a cytotoxic protein with a cytolytic effect on erythrocytes, epithelial cells, PMNs, monocytes, mast cells, basophils and lymphocytes. By lysing erythrocytes, Hly provides bacteria access to iron [11]. Moreover, Hly induces a pro-inflammatory response by interacting with voltage-gated Ca^2+^ channels on cell membranes and activating nuclear factor kappa-light-chain-enhancer of activated B cells (NF-κB), leading to increased production of pro-inflammatory cytokines such as interleukin (IL)−6 and IL-8 [12]. Bacterial dissemination is facilitated by Hly-mediated calcium entry, which activates caspase-dependent pathways, leading to tubular cell apoptosis and disruption of cellular junctions [12]. Furthermore, Hly triggers the NACHT leucin-rich repeat PYD protein 3 (NLRP3) inflammasome in a potassium-dependent manner, leading to macrophage death through pyroptosis [12, 14].

Another UPEC virulence mechanism involves the secretion of Toll/interleukin-1 receptor (TIR) domain containing-protein C (TcpC). It interferes with Toll-like receptor (TLR) signalling (preventing NF-κB activation), binds to myeloid differentiation factor 88 (MyD88), and suppresses NLRP3 inflammasome activation, as well as the production of ROS and pro-inflammatory cytokines induced by LPS [13].

Although *E. coli* is the predominant cause of fUTI, other bacteria can contribute, particularly in patients with predisposing factors. The normal flow of urine is the first line of defence of the urinary tract against pathogens, preventing bacteria attachment to the urothelium. Conditions that disrupt normal urine flow—such as CAKUT, neurogenic or dysfunctional bladder, detrusor instability, and constipation—can increase the risk of developing fUTIs [1].

When a bacterial infection reaches the kidney, the innate immune response triggers localized inflammation. If this inflammation is promptly treated and remains transient, the kidney parenchyma can recover fully [15].

#### Innate immune response

**Fig. 2:**
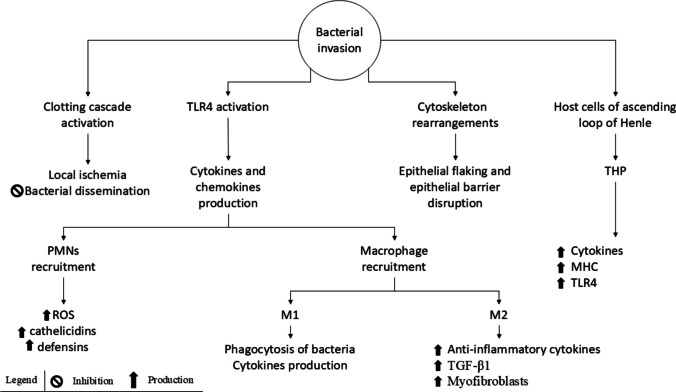
Innate immune response. Bacterial invasion initiates a cascade of defence mechanisms aimed at halting the infection. Activation of the clotting cascade helps physically obstruct bacterial invasion. TLR4 recognizes LPS, P fimbriae, and type 1 fimbriae on the surface of UPEC, triggering the production of pro-inflammatory cytokines and chemokines. These signalling molecules recruit PMNs and macrophages to the site of infection. These immune cells release toxic enzymes, phagocytose the bacteria, and produce additional pro-inflammatory cytokines to amplify immune response. Following kidney epithelial invasion, UPEC induces cytoskeletal rearrangement, leading to epithelial flaking and disruption of the epithelial barrier function. Additionally, cells in the ascending loop of Henle produce THP, which plays a multifaceted role in the immune response. THP stimulates cytokine production, upregulates MHC expression on DC, and activates the TLR4 pathway. Major histocompatibility complex, MHC; polymorphonuclear, PMN; reactive oxygen species, ROS; Tamm-Horsfall protein, THP; Toll-like receptor, TLR; transforming growth factor beta, TGF-β

The invasion of the kidney epithelium and subsequent local tissue damage by UPEC triggers a cascade of events that unfold within 3 to 4 h (Fig. 2). Tubuloepithelial cells undergo significant cytoskeletal rearrangements, resulting in the malfunction of intercellular junctions, epithelial shedding, and disruption of the epithelial barrier [12].

Before the infiltration of PMNs, the activation of the clotting cascade serves as an early protective mechanism, forming a physical barrier to bacterial dissemination. The resulting clot formation causes localized ischemia in the infected nephron, trapping UPEC in the proximal tubule lumen and preventing endothelial invasion while other defence mechanisms are mobilized [12].

The innate immune response is activated through the Toll-like receptor (TLR) signalling cascade. The primary TLR expressed by uroepithelial cells is the TLR4, which recognizes the LPS, P fimbriae, and type 1 fimbriae on UPEC surfaces. Activation of TLR4 triggers a pro-inflammatory response, leading to the production of cytokines such as type 1 interferon (IFN-1), IL-6, tumor necrosis factor (TNF), as well as chemokines like IL-8, C–C motif chemokine ligand 3 (CCL), CCL5 (also known as RANTES), and monocyte chemoattractant protein-1 (MCP). These mediators orchestrate the recruitment and infiltration of inflammatory cells [12, 13]. Among the inflammatory cells, PMNs are the first to be recruited in the infection site. They phagocytose and destroy bacteria but also contribute to tissue damage through the release of toxic enzymes from their granules [12, 13]. Phagocytosis is initiated when PMN complement receptors bind the bacteria opsonized by the complement proteins. The bacteria are internalized into phagosomes, where a respiratory burst generates ROS that are lethal to UPEC [9]. Polymorphonuclear cells recognise IL-8 through the CXC motif chemokine receptor 1 (CXCR) and CXCR2, migrate into the kidney interstitium by binding intracellular adhesion molecule 1 (ICAM1), and secrete metalloproteinases to degrade the tubular basement membrane, allowing them to reach the urinary lumen. Macrophages are recruited shortly after PMNs via CCL2 binding to its receptor. They differentiate into two subsets: M1 macrophages, which support the pro-inflammatory phase by producing cytokines and phagocytosing bacteria, and M2 macrophages, which promote the resolution phase and tissue repair by producing anti-inflammatory cytokines and growth factors [10].

Host cells produce antimicrobial peptides with both direct and indirect antimicrobial effects, including Tamm-Horsfall protein (THP), defensins, and cathelicidins. The THP, a glycoprotein produced by cells of the ascending loop of Henle, stimulates cytokine production, upregulates major histocompatibility complex (MHC) expression on dendritic cells (DC), and activates the TLR4 signalling pathway. However, excessive THP activity can contribute to the pathogenesis of interstitial nephritis [12].

Defensins and cathelicidins, produced by infiltrating PMNs and local kidney epithelium, exhibit direct antimicrobial activity. They also facilitate immune cell recruitment, promote inflammation, and enhance bacterial opsonization [12, 13].

#### Effects of the innate immune response on kidney

The innate immune response plays a critical role in combating urinary tract infection (UTI). However, some mechanisms that effectively eradicate the infection can also inflict significant damage on kidney tissues. Various immune cells contribute to this process, including PMNs, which produce ROS through a pathway mediated by Rho family guanosine triphosphatases that activate nicotinamide adenine dinucleotide phosphate (NADPH) oxidase. While ROS are highly effective in killing UPEC, they also contribute to host tissue destruction and are implicated in the development of kidney damage, fibrosis, and eventually CKD.

M2 macrophages also produce ROS along with matrix metalloproteinase-9 (MMP9), which activate transforming growth factor beta 1(TGF-β1) and myofibroblasts. This activation plays a key role in the induction of kidney fibrosis [10].

### Acute parenchymal involvement

When discussing acute parenchymal involvement, the primary focus is on acute pyelonephritis, the most common form of such involvement [1]. One of the most sensitive tools for detecting kidney parenchymal involvement during fUTI is the dimercaptosuccinic acid (DMSA) scan [16, 17]. This imaging technique is effective in identifying both the acute phase of pyelonephritis and chronic damage, such as kidney scarring. Approximately 85% of children with fUTIs show decreased uptake on DMSA scan performed during or shortly after the infection [16]. Of these, 10 to 40% will develop permanent kidney scarring, irrespective of age [15]. Recent evidence, however, indicates that kidney scarring following fUTI is focal and does not result in severe impairment of kidney function, unlike diffuse congenital (hypo-)dysplasia, which represents a major risk factor for progressive CKD [8, 18].

Although the DMSA scan is considered the gold standard for confirming acute pyelonephritis during fUTI, its routine use is not recommended due to radiation exposure. Instead, diagnosis is typically based on clinical signs and laboratory findings [1]. Febrile UTI should be suspected in cases of fever ≥ 38 °C without an identifiable source [19, 20]. In infants aged 2 to 3 months, fever may be absent, and symptoms such as lethargy, irritability, and vomiting may predominate [21]. Importantly, the absence of fever in infants under 3 months does not indicate a milder condition [21]; rather, the risk of complications such as sepsis and meningitis is higher in this age group [22]. In older children, symptoms such as frequent or painful urination and changes in continence may be early signs, while abdominal pain and lower back tenderness are often associated with fever [19]. The presence of leucocytes, with or without nitrites, can support the clinical suspicion of fUTI, while a positive urine culture with the growth of a single bacterial species from a properly collected sample confirms the diagnosis [19, 20, 23].

Several readily available laboratory tests, such as elevated white blood cell (WBC) counts and C-reactive protein (CRP) levels, have been evaluated as indicators of renal involvement. However, their specificity and sensitivity remain low [1]. A 2020 Cochrane review sought to identify a reliable biomarker to replace the acute DMSA scan for assessing renal parenchymal involvement during UTIs in children. The review compared the diagnostic accuracy of CRP, procalcitonin (PCT), and erythrocyte sedimentation rate (ESR). It concluded that ESR is not effective in differentiating between lower urinary tract infections (LUTIs) and acute pyelonephritis. However, low CRP levels are useful for ruling out acute pyelonephritis, while PCT is more effective in confirming its diagnosis [24].

Many studies have sought to identify new potential markers for UTIs. Among these, the most extensively studied markers include neutrophil gelatinase-associated lipocalin (NGAL), various cytokines, and various chemokines [25].

NGAL is produced by epithelial cells in the loop of Henle, collecting tubules, bladder urothelium, and immune cells (PMN, macrophages) in response to inflammatory triggers. During a UTI, NGAL is released into the urine, where it inhibits bacterial replication by sequestering iron. Due to this role, both serum and urinary NGAL have been explored as potential biomarkers for detecting UTIs and distinguishing LUTIs from acute pyelonephritis [25]. In 2024, Mattoo et al. analyzed recent systematic reviews and meta-analyses to evaluate the utility of emerging UTI biomarkers in children, including NGAL. Their analysis highlighted NGAL as a promising diagnostic marker for UTIs, but its ability to differentiate LUTIs from acute pyelonephritis remains uncertain [25]. Moreover, a deeper understanding of NGAL’s biological function, along with the development of standardized testing methods, is crucial to fully establish its diagnostic potential [25].

Other promising biomarkers for UTIs include IL-6 and IL-8. A 2022 meta-analysis by Hosseini et al. reported that serum levels of IL-6 and IL-8 in children with febrile UTIs were not significantly different from those in children with febrile conditions unrelated to UTIs [26]. However, urinary levels of IL-6 and IL-8 were significantly higher in children with fUTIs compared to those with other febrile illnesses [26]. Unfortunately, optimal cutoff values for these biomarkers have yet to be determined [26].

Additional biomarkers showing promise include IL-1β, CXCL1, alpha defensin 5, human neutrophil peptides 1–3, and heat shock protein 70, all of which have been found at elevated urinary levels in children with UTIs [25]. These findings warrant further investigation to establish their diagnostic utility [25].

When fUTI is suspected based on clinical and laboratory findings, prompt initiation of empirical antibiotic therapy is essential, guided by local bacterial resistance patterns [1].

While most fUTIs resolve without complications, their severity depends on factors such as urinary tract malformations, the timeliness of treatment initiation, and host–pathogen interactions.

Although acute pyelonephritis is the predominant form of kidney parenchymal involvement in fUTI, more severe forms, such as acute focal bacterial nephritis (AFBN, also called acute lobar nephronia), kidney abscess, and pyonephrosis, may occasionally develop [27].

Clinically, these severe forms should be suspected when fever persists for more than three 3 days despite appropriate antibiotic therapy, especially in patients with a history of urological abnormalities [19, 28].

In a 2014 study by Bitsori et al., medical records of 602 children hospitalized for UTI at Heraklion University Hospital from 2003 to 2012 were reviewed to identify cases of complicated infections and assess their characteristics and outcomes [28]. Among these children, 21 developed AFBN, one developed a kidney abscess, and three developed pyonephrosis. Acute focal bacterial nephritis progressed to abscess in two cases, while AFBN and pyonephrosis coexisted in three patients. Most of the complicated cases (*n* = 18) occurred in children with a history of urological abnormalities.

All 25 children with complicated infections presented with fever and clinical deterioration at admission. Laboratory findings showed leucocytosis, elevated CRP, and ESR. Urine cultures identified *E. coli* in 12 cases, *Pseudomonas aeruginosa* in eight cases, *Klebsiella pneumoniae* in three, and *Proteus mirabilis* and *Providencia stuartii* in one case each. One patient had a sterile culture. Blood cultures were positive in all pyonephrosis cases (caused by *P. aeruginosa*) and in one AFBN case.

Kidney ultrasound (US) performed during the acute phase revealed suggestive lesions in 17 of 27 cases, including focal hyper- or hypoechogenic lesions, or nephromegaly.

In cases of persistent fever beyond 72 h of antibiotic treatment, further imaging with computed tomography (CT) or magnetic resonance urography confirmed the diagnosis in five and in 20 cases, respectively. Both imaging modalities are highly accurate in characterizing kidney lesions. All patients received intravenous antibiotics for a median duration of 13 days, followed by oral antibiotics for a median of 21 days. Despite treatment, kidney scarring was reported in 16 of the 25 cases [28].

#### Acute focal bacterial nephritis

Acute focal bacterial nephritis is a rare, localized inflammatory bacterial infection of the kidney, characterized by the presence of an inflammatory mass without drainable pus. It is considered an intermediate condition between acute pyelonephritis and kidney abscess, representing an early stage of the latter. Its pathogenesis is linked to either hematogenous infection or ascending infection from the lower urinary tract, often facilitated by urinary tract malformations such as vesicoureteral reflux (VUR) [27].

**Fig. 3:**
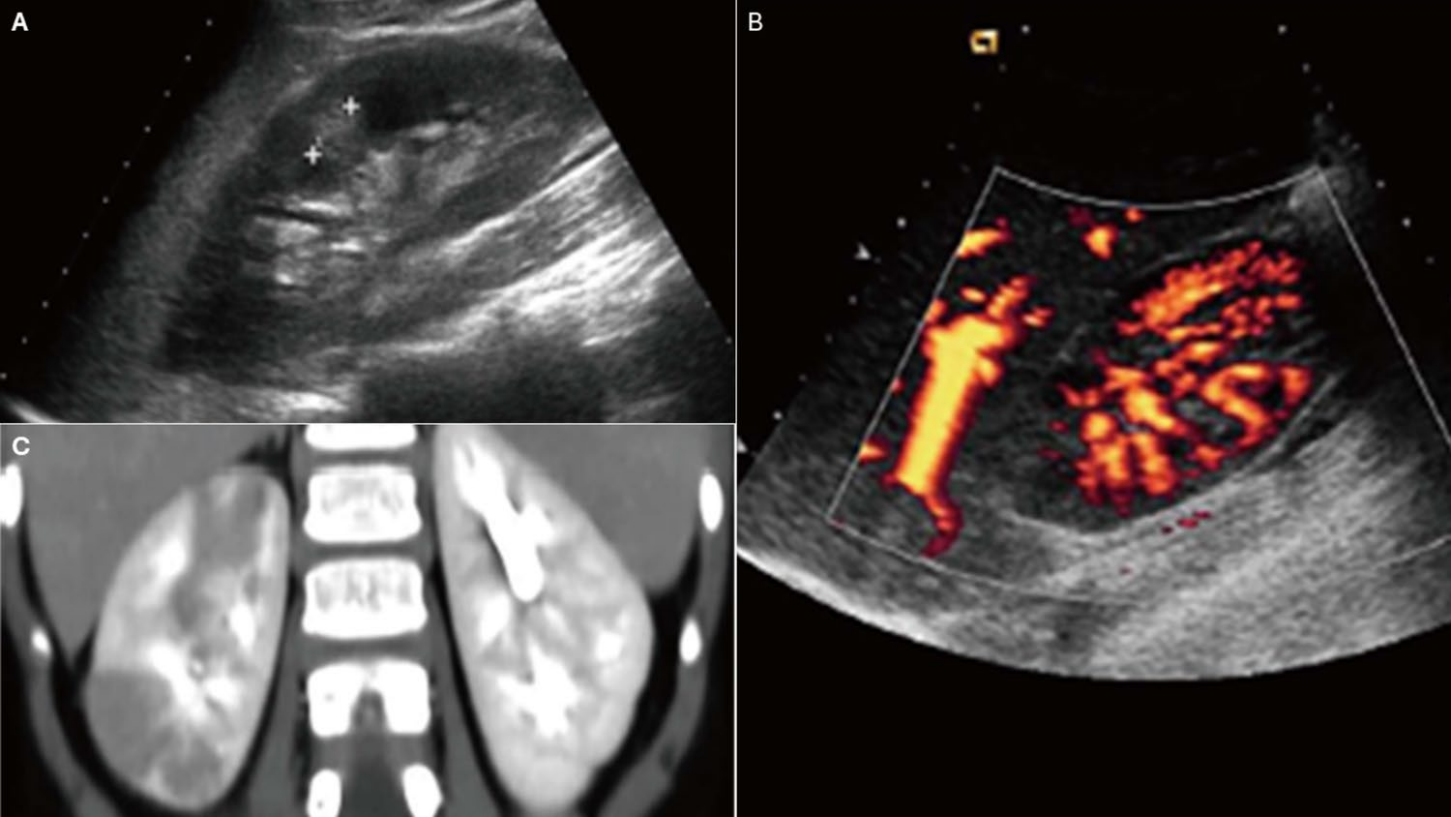
Radiological images of AFBN. **A** Abdominal US reveals a hyperechoic mass in the mid-zone of the kidney. **B** Abdominal US with power-Doppler analysis shows hypoperfused areas in the upper pole of right kidney. **C** Contrast-enhanced CT scan of the abdomen reveals multiple hypodense, non-enhancing lesions in the right kidney. Adapted from refence [30], reproduced under Creative Commons License CC BY. Ultrasound, US; computed tomography, CT

The nonspecific symptoms of AFBN make it challenging to distinguish from acute pyelonephritis, complicating the differential diagnosis between these two conditions. Common symptoms of AFBN include fever, nausea, vomiting, flank, and abdominal pain [29]. Laboratory findings typically show elevated levels of CRP, ESR, and WBC. However, urinalysis and urine culture may not always reveal clear signs of infection [30]. Histologically, AFBN is characterized by interstitial edema, perivascular leukocytic infiltration, and the absence of drainable pus. Diagnosis relies heavily on radiological imaging. US is often the initial screening method, revealing features such as hypo-, iso-, and hyperechogenicity. Doppler US can further aid diagnosis by demonstrating reduced focal blood flow at the lesion site (Fig. 3) [29]. CT scans provide greater detail, showing lobar inflammation and hypodense, wedge-shaped lesions following contrast administration (Fig. 3) [27, 29]. Magnetic resonance imaging (MRI), used less frequently, demonstrates hypointense lesions on T2-weighted images and decreased enhancement on post-contrast T1-weighted images. In a 2017 study by Sieger et al., US demonstrated a sensitivity of 91–96% for detecting AFBN, although it occasionally produced false-positive or false-negative results [29]. CT and MRI are considered superior diagnostic tools, with CT regarded as the diagnostic gold standard.

There is no standardized treatment protocol for AFBN. Antibiotic therapy should be empirically targeted at Gram-negative bacteria, with *E. coli* being the most commonly isolated pathogen. Intravenous antibiotics should be administered until 2–3 days after fever resolution, followed by oral antibiotics for at least an additional 2 weeks [29, 30].

#### Kidney abscess

Kidney abscess is a focal, encapsulated purulent cavity within the kidney parenchyma, histologically characterized by tissue necrosis with liquefaction in the infected area [27]. It is a rare clinical entity in pediatric patients, often misdiagnosed, and its management lacks standardization. Most cases reported in the literature are secondary to UTIs; although, a few result from hematogenous spread. *E. coli* is the most frequently isolated pathogen, followed by *S. aureus* and other Gram-negative bacteria.

The symptomatology of kidney abscesses is nonspecific. Commonly reported symptoms include fever, flank or abdominal pain, vomiting, and, occasionally, UTI-associated symptoms [25–27]. Laboratory findings often show elevated WBC, CRP, ESR, and PCT levels. Urinalysis may reveal leukocyte esterase or nitrites, while blood and urine cultures can help identifying the infection source, with UTIs being the primary trigger [31, 32].

**Fig. 4:**
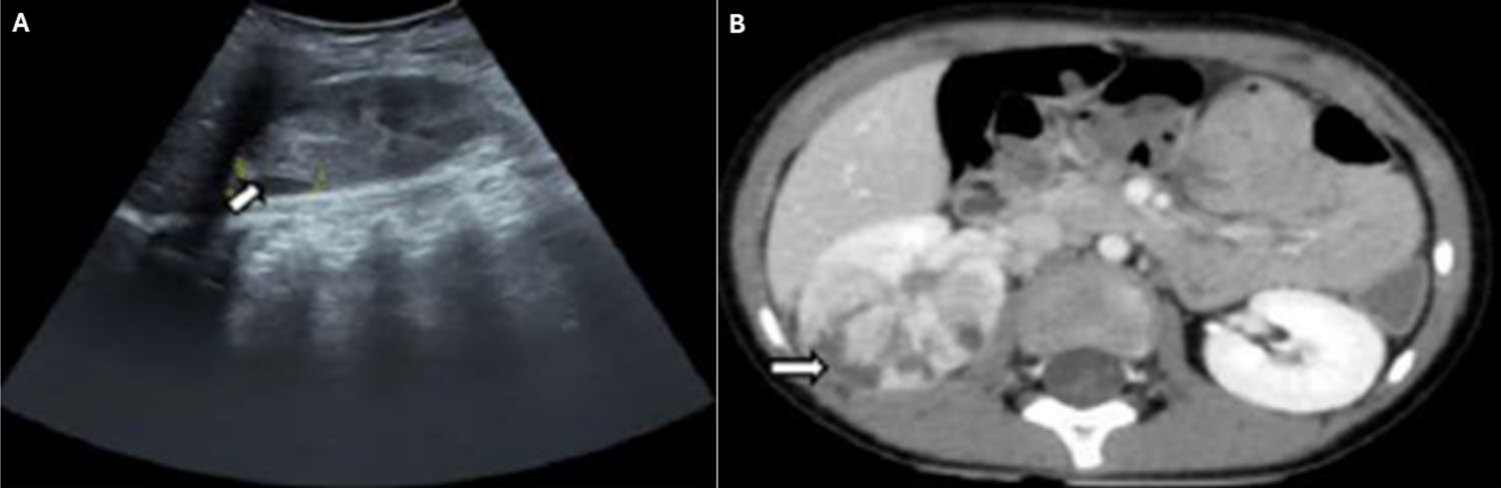
Radiological images of kidney abscess. **A** US reveals a well-defined hypoechoic lesion (arrow). **B** Enhanced CT scan displays a well-defined, low-density mass in the upper right kidney, suggestive of a kidney abscess (arrow). Adapted from reference [31], reproduced under Creative Commons License CC BY. Ultrasound, US; computed tomography, CT

Diagnosis requires radiological confirmation. US typically reveals a well-defined hypoechoic lesion with internal echoes, while CT demonstrates a low-density, well-defined mass with a thick, irregular wall that enhances after contrast administration (Fig. 4) [27, 33]. In 2015, Linder et al. [33] reviewed data from 16 pediatric patients diagnosed with kidney abscess between 1990 and 2012. Of these, 13 cases were secondary to UTIs, one resulted from hematogenous spread due to a skin infection, and two were of unknown etiology. E*. coli* was the most commonly identified pathogen, followed by *S. aureus*. Treatment was guided by therapeutic strategies extrapolated from adult cohort studies, which recommend conservative measures for abscesses < 3 cm. All patients received antibiotics. Among the 11 patients with abscesses < 3 cm, ten were successfully managed conservatively, with only one requiring percutaneous drainage. Of the five patients with abscesses > 3 cm, three underwent successful percutaneous drainage, while the remaining two (with abscesses measuring 3.5 and 4 cm) were successfully treated conservatively.

Based on these outcomes, the authors recommend percutaneous drainage for lesions > 3 cm, persistent fever despite specific antibiotic therapy, and in immunocompromised or critically ill patients. Antibiotic therapy was continued until radiographic resolution of the lesion. During follow-up, voiding cystourethrography (VCUG) was performed in ten patients, revealing grade I VUR in two cases [33]. In contrast, Seguias et al. reported genitourinary abnormalities in 11 out of 36 patients (31%) with kidney abscess, including three with VUR, three with structural bladder abnormality, two with a duplicated collecting system, two with neurogenic bladder, one with posterior urethral valves, one with ureteropelvic junction obstruction, and one with urethral stenosis [32].

Despite variations in literature, children with kidney abscesses should be evaluated for predisposing anatomical or functional genitourinary conditions, such as abnormal ureters or bladder, VUR, kidney dysplasia, posterior urethral valves, and other urological anomalies [31, 32].

The optimal duration of antibiotic therapy for pediatric kidney abscesses has not been established. However, current recommendations suggest a 4–6-week course, even in cases requiring surgical intervention [31].

#### Pyonephrosis

Pyonephrosis is characterized by the accumulation of pus within a hydronephrotic, obstructed kidney. The clinical presentation ranges from asymptomatic bacteriuria to urosepsis [34].

**Fig. 5:**
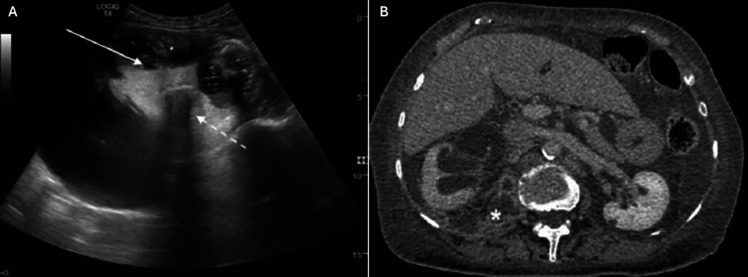
Radiological images of pyonephrosis. **A** US shows hydronephrosis and cortical thinning with high-grade dilation of the kidney collecting system filled with fluid and hyperechoic debris. **B** Axial CT imaging following intravenous contrast administration reveals hydronephrosis and thickening of the kidney pelvic wall. The perirenal space contains fluids, and an abscess is observed in the ileopsoas muscle. Adapted from reference [35], reproduced under Creative Commons License CC BY. Ultrasound, US; computed tomography, CT

Diagnosis is typically established using US, which reveals hydronephrosis with hyperechoic debris in the collecting system (Fig. 5). In some cases, gas in the collecting system may be indicated by large hyperechoic lines producing “dirty” acoustic shadows. CT scan provides additional details, showing hydronephrosis along with parenchymal or perinephric inflammatory changes, gas–fluid or fluid–fluid levels in the kidney collecting system, and thickening of the kidney pelvic wall (Fig. 5) [35].

Information on pyonephrosis is limited and primarily derived from case reports. In 2014, Bitsori et al. identified six cases of pyonephrosis among 602 children hospitalized for fUTI. In three cases, pyonephrosis coexisted with AFBN. In the remaining three cases, where pyonephrosis occurred alone, blood cultures were positive for *P. aeruginosa*. In all cases, US was sufficient to suggest the diagnosis.

All children with pyonephrosis had underlying urinary tract anomalies, and two required nephrectomy or hemi-nephrectomy [28]. Additionally, all patients underwent corrective surgery following the acute phase [28].

The treatment of pyonephrosis involves both managing the infection and addressing the underlying obstruction through corrective surgery. Percutaneous nephrostomy is considered as a safe and highly effective procedure [34].

#### Other forms of “acute” parenchymal kidney involvement

Xanthogranulomatous pyelonephritis (XPN) is an uncommon form of chronic inflammatory kidney disease characterised by destruction of kidney parenchyma, which is replaced by fatty nodules that sometimes contain central necrosis [36]. The precise pathogenic mechanism underlying XPN is poorly understood. However, contributing factors include chronic urinary tract obstruction, inadequately treated infections, lipid metabolism disorders, and altered immune response [36]. In children, urinary tract malformations and obstruction caused by calculi are often associated with XPN development [36].

Primary symptoms include flank pain, intermittent fever, anorexia, weakness, malaise, weight loss, and, occasionally, a palpable abdominal mass [36]. Laboratory findings typically show elevated ESR and PCR, leucocytosis, and microcytic anemia. Urinalysis often indicates pyuria, hematuria, and proteinuria. Notably, only 70% of patients exhibit positive urine cultures, most commonly involving *E. coli* and *P. mirabilis* [36]. There are two forms of XPN: the diffuse form and the focal (or pseudo-tumoral) form, with the latter being more common in pediatric age [36].

**Fig. 6:**
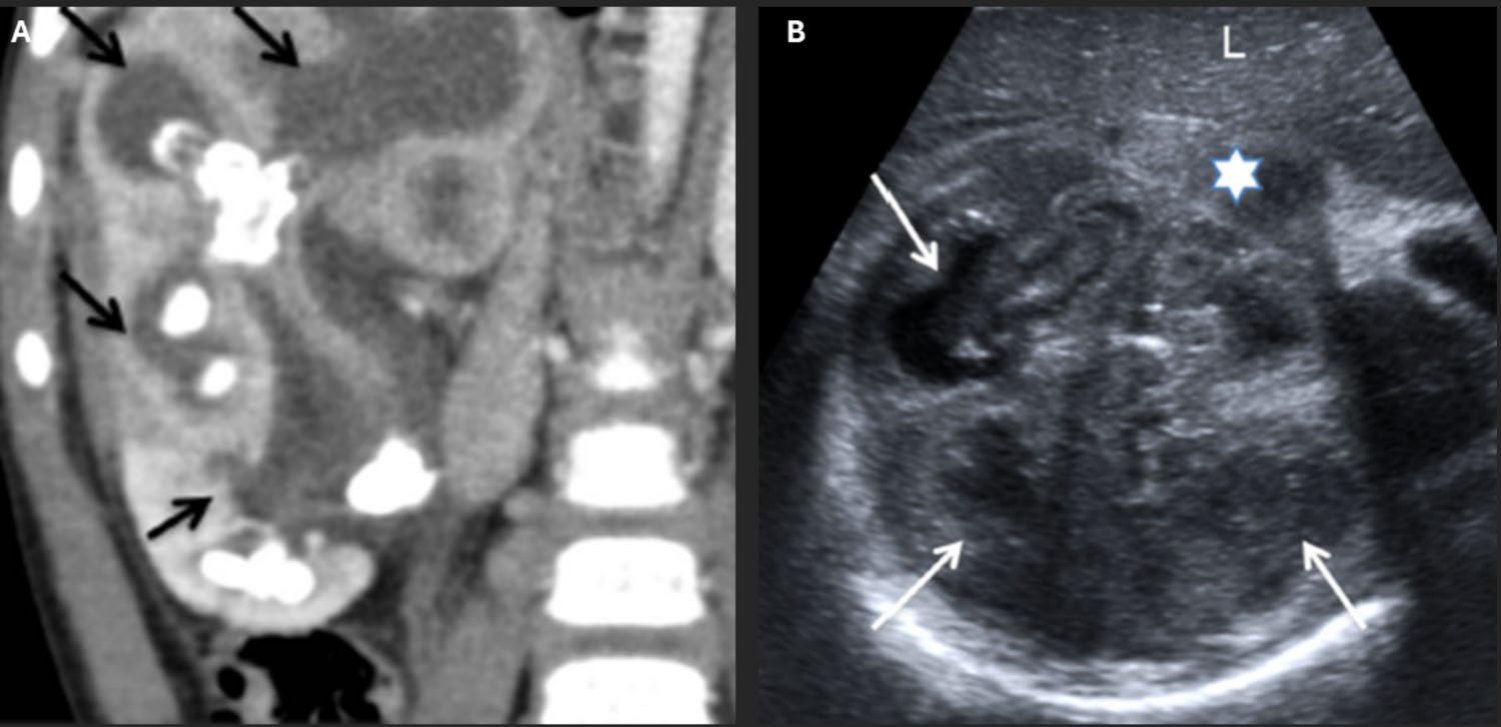
Radiological images of XPN. **A** Diffuse XPN. CT scan after intravenous contrast administration shows hypodense round areas (arrows) in the right kidney, accompanied by calculi in the calyces. **B** Localized XPN. US shows multiple hypoechoic masses (arrows) replacing normal parenchyma of the right kidney, along with the presence of perinephric fluid (star). Adapted from reference [36], reproduced under Creative Commons License CC BY. Xanthogranulomatous pyelonephritis, XPN; ultrasound, US; computed tomography, CT

Focal XPN is confined to a specific kidney segment or one pole of a duplex system. US and CT are valuable diagnostic tools, though distinguishing between XPN, kidney abscess, and neoplasm can be challenging [36]. On US, a localized hypoechoic mass may be observed (Fig. 6). CT scans reveal a well-defined, localized intrarenal mass with water-like attenuation and rim enhancement. Magnetic resonance imaging shows an isointense lesion on T1-weighted images and a slightly hypointense signal on T2-weighted images [36]. Biopsy is essential for distinguishing focal XPN from kidney tumors. Management options include partial nephrectomy, drainage, and antibiotic therapy [36].

Diffuse XPN appears on US as an enlargement of the whole kidney with preserved reniform shape, hypoechoic areas are visible in correspondence of calyceal dilatation and parenchymal destruction. Lithiasis is a frequent finding (83% of XPN cases) [36]. CT imaging shows kidney enlargement with multiple hypodense, round areas replacing kidney parenchyma. After contrast administration, rim enhancement of the cavities is observed (Fig. 6), and lipomatous components may aid in diagnosis [36]. The kidney pelvis often contains a central calculus, and multiple calculi may be present within the calyces. MRI findings include isointense images on T2-weighted scans compared to the normal kidney tissue, hypointense cavity contents on T1-weighted scans, and hyperintense cavity contents on T2-weighted scans displaying fluid–fluid levels [36]. Perirenal infiltration appears hypointense on both T1- and T2-weighted images, likely due to fibrinous exudate. In diffuse XPN, clinical evaluation and imaging are sufficient for diagnosis, rendering biopsy unnecessary. Treatment typically involves total nephrectomy, particularly when kidney function is approximately 20%, often combined with antibiotic therapy [36].

Milk of calcium refers to a viscous colloidal suspension of calcium salts that forms within cysts or other body cavities, such as kidney cysts and the urinary tract [37]. While the exact etiology is unclear, factors such as urine stagnation, obstruction, and infection are thought to contribute [37]. Significative clinical signs include milky urine and the expulsion of soft, whitish material, which is pathognomonic [37]. On US, milk of calcium appears as an echogenic focus with an acoustic shadow that shifts with changes in patient position [37]. CT scans reveal a crescent-shaped dense pattern.

Management aims to preserve the kidney function. Nephrectomy is reserved for cases with no residual kidney function, while percutaneous nephrostomy is an effective treatment option [37]. Some authors advocate stent placement, even in cases of severely impaired kidney function, to evaluate potential recovery. This approach was successfully employed in a toddler with milk of calcium, whose kidney function improved from 0% (based on mercapto-acetyl-tri-lycine scintigraphy) to 18% after 1 month [37].

### Chronic parenchymal sequelae

**Fig. 7:**
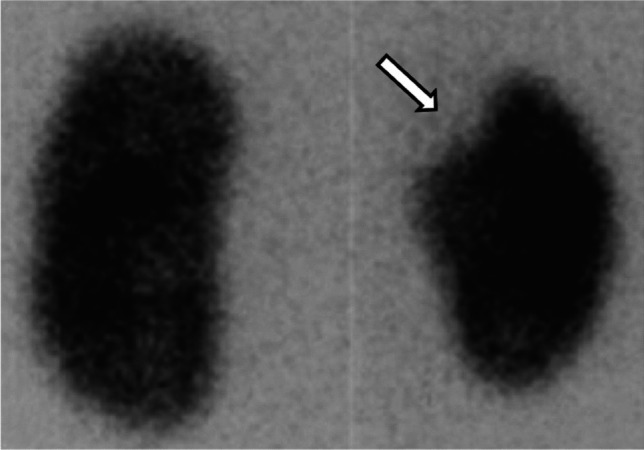
Kidney scar presenting as a photopenic region on the upper pole of right kidney (arrow) on DMSA scan. Figure modified from reference [38], reproduced under Creative Commons License CC BY. Dimercaptosuccinic acid, DMSA

As previously discussed, the inflammatory response plays a crucial role in clearing the infection. However, when the inflammation becomes uncontrolled, functional kidney parenchyma can be replaced by extracellular matrix, leading to kidney scarring. The most accurate diagnostic tool for detecting kidney scars is DMSA scan, which identifies scars as photopenic regions (Fig. 7) [10].

In 2019, Shaik et al. [39] conducted an observational study to evaluate the association between the number of fUTIs and the risk of developing kidney scars. The study utilized data from two multicenter studies: RIVUR (Randomized intervention for children with vesicoureteral reflux) and CUTIE (careful urinary tract infection evaluation). These studies followed children with UTIs and no known genitourinary malformations for 2 years. All participants underwent DMSA scans at baseline and at the end of the follow-up to assess for kidney scarring. The results showed that the incidence of kidney scarring in children was 0% after one non-febrile UTI, 2.8% after one fUTI, 25.7% after two fUTIs, 28.6% after three or more fUTIs. These findings indicate that the risk of kidney scarring increases significantly after the second fUTI, underscoring the importance of strategies to identify children at higher risk of recurrent infections [39].

In 2016, another observational study using RIVUR and CUTIE data identified delayed initiation of antibiotic therapy as a risk factor for kidney scarring. Delay was defined as the time between fever onset and the start of antibiotic treatment. Data showed that children who did not develop kidney scars had an average fever duration of 48 h, while those who developed scars experienced an average fever duration of 72 h [2].

These findings emphasize the need to integrate effective strategies into clinical practice to promptly diagnose and treat UTIs while preventing recurrence. For early detection, it is critical to avoid delays in urine testing. To prevent additional episodes, it is essential to identify patients requiring further investigations based on recommendations [19, 40–43] and to address modifiable risk factors in toilet-trained children, such as retentionist urination habits, low water intake, and constipation [44].

It is also important to note that congenital kidney dysplasia, often associated with CAKUT, plays a significant role in the development of scars or nephropathy.

The PREDICT study [18] provided valuable insights into the link between kidney scarring and congenital dysplasia associated with CAKUT. This randomized, open-label trial included 292 children aged 1 to 5 months with grade III, IV, or V VUR and no prior UTI. Participants were randomized to receive either continuous antibiotic prophylaxis or no treatment for 2 years.

The study’s primary outcome was the occurrence of the first UTI during the trial, while the secondary outcomes included evaluating the effects of antibiotic prophylaxis on kidney scarring and estimated glomerular filtration rate (GFR). Results showed that the incidence of new kidney scarring on DMSA scan were similar between children receiving antibiotic prophylaxis and those not receiving it, regardless of fUTI occurrence [18]. Moreover, the estimated GFR at 24 months was comparable in children with and without fUTIs, suggesting that CAKUT did not increase the risk of more severe kidney damage in the short term, even after acute pyelonephritis [18].

#### How can scar formation be prevented?

The probability that a child with a fUTI will develop kidney scars ranges from 10 to 40%, even with adequate antibiotic therapy. These scars may lead to long-term complications such as hypertension and chronic kidney damage, particularly in children with underlying kidney disease or congenital kidney dysplasia. To identify effective strategies for reducing the risk of kidney scarring, several randomized controlled trials have investigated the use of corticosteroids as an adjunct to antibiotic therapy, based on the hypothesis that inflammation is the primary driver of kidney damage [45].

In 2020, Shaikh et al. conducted a randomized trial involving 385 children aged 2 to 6 years with their first episode of fUTI. Participants were randomized to receive either a placebo or oral corticosteroids (dexamethasone 0.15 mg/kg/dose twice daily) for 3 days, in addition to a 10-day course of appropriate antibiotics. Kidney scarring was assessed using DMSA scans 5–24 months after enrolment. The study found that 9.8% of children receiving corticosteroids developed kidney scars, compared to 16.8% of those in the placebo group. However, the difference was not statistically significant. The study also observed that children aged 2 years or older were at higher risk of developing scars [46].

In 2021, Da Dalt et al. conducted a similar, smaller multicenter randomized controlled trial involving 48 children aged 2 to 24 months. Participants were randomized to receive either oral dexamethasone (0.15 mg/kg/dose twice daily) for 4 days or a placebo, alongside a 10-day antibiotic course. Due to the small sample size, a Bayesian analysis was performed, revealing a 50% probability that corticosteroid therapy could reduce kidney scarring by 20% [45].

In 2023, Gkiourtzis et al. conducted a meta-analysis to synthesize existing evidences on the use of corticosteroids for preventing kidney scarring in children with acute pyelonephritis. The studies included in the meta-analysis varied in corticosteroid type, duration of therapy (3–4 days), and mode of administration. A total 693 patients met the inclusion criteria. Despite the heterogeneity of the sources, the meta-analysis found a statistically significant reduction in kidney scarring among patients who received adjunct corticosteroids with antibiotic treatment (relative risk 0.64, 95% confidence interval 0.42–0.98, *p* = 0.04). Importantly, corticosteroid use was not associated with prolonged hospitalization, bacteriemia, or recurrent UTIs [47].

While these findings are promising, further well-designed clinical trials are needed to confirm the efficacy and safety of corticosteroids in reducing kidney scarring after acute pyelonephritis before they can be strongly recommended for routine clinical use.

Another potential strategy to prevent kidney scarring is continuous antibiotic prophylaxis. However, as noted earlier, the PREDICT study did not demonstrate any significant effect of continuous prophylaxis in preventing kidney scars [18].

## Tubulo-interstitial involvement

Studies indicate that pediatric inpatients with fUTIs often experience imbalances in sodium (Na +), potassium (K +), chloride (Cl −), acid–base balance, or water homeostasis [3, 48–53]. These disturbances are particularly common in newborns and infants with dilating urinary tract malformations [3]. Tubulo-interstitial involvement in these cases can manifest at three levels: impaired urinary excretion of acids, reduced ability to concentrate urine, and the development of secondary transient pseudohypoaldosteronism (TPHA).

### Impaired urinary excretion of acids

In 1986, Tulassay et al. [54] identified a reduction in urinary pCO_2_ in maximally alkaline urine among children with fUTI as a potential marker of impaired H^+^ secretion in the distal nephron [54]. Unfortunately, this area of research has not been further explored.

### Reduced ability to concentrate urine

The association between kidney parenchyma involvement in acute UTIs and a mild reduction in maximal urinary concentrating ability is traditionally considered an early indicator of bacterial invasion into the kidney medulla [3, 55–57]. Affected children may present with normal or high-normal blood Na^+^ levels. It is hypothesized that the decreased urinary concentrating ability in infants with infectious kidney parenchymal involvement may result from increased medullary blood flow, which diminishes the osmotic gradient between the outer and inner medulla. Alternatively, this reduction could be due to impaired water reabsorption in nephron segments upstream of the collecting duct [3]. However, studies documenting reduced concentrating ability face interpretative challenges. Fluid restriction to evaluate concentrating capacity in the context of fUTI would be clinically inappropriate. Moreover, an increased output of dilute urine is often observed in patients receiving intravenous fluids, further complicating assessment.

### Secondary transient pseudohypoaldosteronism

Secondary transient pseudohypoaldosteronism is characterized by hyponatremia, hyperkalemia, low urinary K^+^ excretion, metabolic acidosis with normal anion gap, fluid volume depletion, and impaired kidney function. This condition predominantly affects boys (approximately 80% of cases) within the first year of life, particularly those with UTIs and genitourinary tract malformations. It is marked by tubular unresponsiveness to aldosterone, leading to massive activation of the renin–angiotensin–aldosterone system [3, 58, 59]. Secondary transient pseudohypoaldosteronism has also been linked to AKI during fUTI [58, 59]. The mechanisms underlying aldosterone under-responsiveness during infancy in the context of UTIs are not fully understood. Three primary hypotheses have been proposed: (a) an inflammatory response, potentially involving transforming growth factor-β, may play a role; (b) differences in kidney structure and function between infants and older children could contribute; (c) genetic predispositions, similar to dominant primary pseudo-hypoaldosteronism, might occasionally influence secondary cases [59].

Early but non-specific symptoms that should raise suspicion of secondary TPHA in infants with concomitant obstructive uropathy and/or UTI (even in the absence of fever) include poor appetite, vomiting, and failure to gain weight.

Diagnostic evaluation for suspected secondary TPHA involves urinalysis and urine culture to confirm a UTI, serum electrolytes, glucose, cortisol, and urinary sodium. Hyponatremia alongside normal cortisol levels suggests TPHA and helps differentiating it from congenital adrenal hyperplasia, which typically shows reduced cortisol levels and elevated 17-hydroxyprogesterone levels. The diagnosis of TPHA is confirmed by the presence of elevated renin and aldosterone levels.

The trans-tubular potassium gradient (TTKG) is a valuable diagnostic tool for assessing tubular K^+^ handling. Hyperkalemia typically stimulates renin release, leading to an expected increase in tubular K^+^ secretion. In cases where TTKG is less than six in a hyperkalemic child, it indicates impaired aldosterone action.

Treatment of secondary TPHA typically involves fluid administration with isotonic solutions (e.g., 0.9% saline or ringer lactated) in addition to antibiotic therapy [60]. Normal saline is ideal for correcting hyponatremia, hyperkalemia, and fluid deficits, while ringer lactate is preferred for correcting acidosis. The use of hydrocortisone and fludrocortisone is not recommended. Electrolyte imbalances, acidosis, and kidney function generally normalize within 1 week of appropriate treatment, with a good prognosis. Severe cardiac arrhythmias due to hyperkalemia are rare, and no fatalities have been reported [59].

Recently, a study on hospitalized children with fUTIs have identified underlying CAKUT as major risk factor for the developing concomitant hyponatremia and hyperkalemia, suggestive of TPHA [58].

## Acute kidney injury

An Italian multicenter retrospective study analysed data from 849 pediatric patients hospitalized for fUTI to assess the prevalence and risk factors for AKI in this population. Among the cohort, 124 (14.6%) were diagnosed with AKI. The study identified high neutrophil levels and the presence of CAKUT as the two most significant predictors of AKI. Notably, 285 of the 849 children had a form of CAKUT, and 89 (31.2%) of these developed AKI. This prevalence may be attributed to the reduced nephron mass associated with CAKUT, which limits the kidney’s ability to sustain function during stressful conditions such as fUTI. Additional risk factors identified included dehydration, systemic inflammation and age > 8 months.

These findings are crucial for predicting the potential progression of children with a history of AKI to CKD and for planning appropriate follow-up. The study emphasized that the development of AKI during fUTI is a reliable indicator of VUR and may inform the decision to perform a VCUG [4].

The prevalence of AKI among children with fUTI is concerning, as AKI significantly increases the risk of CKD. Mammen et al. showed that the incidence of CKD within 1–3 years following an episode of AKI was 10.3%. Additionally, 46.8% of the 126 patients evaluated developed conditions such as hypertension, hyperfiltration, and mildly decreased GFR, placing them at greater risk of CKD over time [61]. Supporting this, a meta-analysis by Coca et al. confirmed that AKI survivors face a markedly increased risk of developing CKD and kidney failure, with the risk rising in proportion to AKI severity. While the exact mechanism behind this association remains unclear, experimental animal studies suggest that AKI can induce kidney fibrosis, contributing to long-term kidney damage [5].

## Conclusions

In conclusion, fUTIs can impact the kidneys at multiple levels, with both acute and chronic effects. Acute involvement, such as AFBN, kidney abscess, and pyonephrosis, may present with non-specific symptoms (fever, nausea, vomiting, and flank pain) and laboratory findings. An acute complication should be suspected if the patient does not respond to antibiotic therapy with defervescence within 72 h or if fever recurs during treatment. In such cases, radiological imaging is essential for accurate diagnosis and management. Chronic kidney sequelae manifest as kidney scarring, which appears as photopenic regions on DMSA scans. Current evidence suggests that corticosteroids may reduce the risk of kidney scarring, while antibiotic prophylaxis does not appear to have significant protective effects.

During an episode of fUTI, the kidneys may also experience tubulointerstitial involvement, resulting in fluid and electrolyte imbalances. This can occur due to a reduced ability to concentrate urine or secondary TPHA, characterized by tubular resistance to aldosterone and uncontrolled activation of the renin–angiotensin–aldosterone system.

Lastly, AKI is another potential consequence of fUTI, with a higher incidence observed in children with concomitant CAKUT. Proper diagnosis of AKI is crucial to identify patients who require long-term follow-up, as they are at an elevated risk of developing CKD and kidney failure.

## Key summary points

1. During a febrile urinary tract infection (fUTI), the kidneys may be affected at multiple levels, including parenchymal complications, tubulointerstitial involvement, and the potential development of acute kidney injury (AKI).
2. Parenchymal complications can manifest as acute conditions, such as pyelonephritis or renal abscess, and as chronic sequelae like scar formation.
3. A lack of clinical improvement after 72 h of appropriate antibiotic therapy should prompt further investigation to rule out significant parenchymal involvement.
4. Infants with congenital anomalies of the kidney and urinary tract (CAKUT) and fUTI are at increased risk of fluid and electrolyte imbalances due to impaired tubulointerstitial function.
5. Children who experience AKI during a fUTI are at higher risk of having underlying CAKUT.

## Multiple choice questions

Answers given following the references.

1. Which one is not a protective mechanism against UTI?
  - Clotting cascade activation
  - TLR signalling
  - TGF-β1
  - IFN-1
2. Which condition leads to higher risk of fUTI?
  - CAKUT
  - Neurogenic bladder
  - Constipation
  - All of the above
3. Which is the correct statement on kidney abscess?
  - The diagnosis requires radiological confirmation
  - Symptoms of kidney abscess are very specific
  - High CPR and WBC are very suggestive of kidney abscess
  - Percutaneous drainage is the only effective treatment
4. Find the correct statement on kidney scars
  - The risk of kidney scars formation is not influenced by delays in initiating antibiotic treatment
  - The risk of kidney scar formation significantly increases with recurrent fUTIs
  - Antibiotic prophylaxis is effective in reducing kidney scar formation in children with VUR
  - The use of corticosteroids has not been shown to reduce the risk of kidney scar formation
5. Which of the following is a manifestation of secondary transient pseudohypoaldosteronism?
  - Hyponatremia
  - Hyperkaliemia
  - Acidosis
  - All of the above

## Supplementary information

Below is the link to the electronic supplementary material.

Graphical abstract(PPTX 442 KB)

## Data Availability

Not applicable.
