# Supplementary material for: Kidney involvement during the course of febrile urinary tract infection
Source: Pediatr Nephrol. 2025 Feb 25;40(8):2455–68. doi: 10.1007/s00467-025-06695-4 (PMC12187806; doi:10.1007/s00467-025-06695-4)
Supplement: Supplementary file 1 — Graphical abstract(PPTX 442 KB) [file 467_2025_6695_MOESM1_ESM.pptx]

## Slide 1
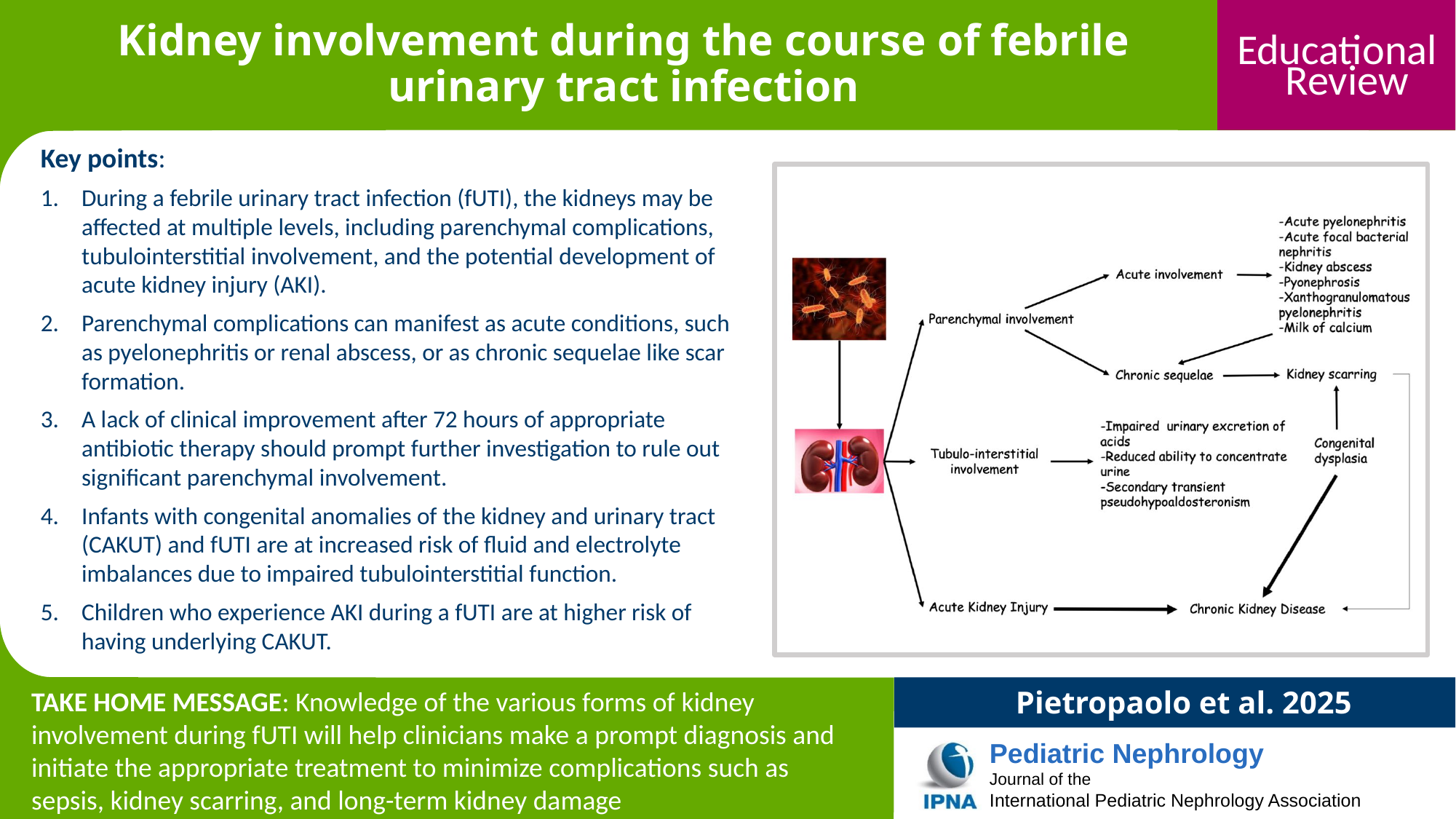

Kidney involvement during the course of febrile urinary tract infection
Key points:
During a febrile urinary tract infection (fUTI), the kidneys may be affected at multiple levels, including parenchymal complications, tubulointerstitial involvement, and the potential development of acute kidney injury (AKI).
Parenchymal complications can manifest as acute conditions, such as pyelonephritis or renal abscess, or as chronic sequelae like scar formation.
A lack of clinical improvement after 72 hours of appropriate antibiotic therapy should prompt further investigation to rule out significant parenchymal involvement.
Infants with congenital anomalies of the kidney and urinary tract (CAKUT) and fUTI are at increased risk of fluid and electrolyte imbalances due to impaired tubulointerstitial function.
Children who experience AKI during a fUTI are at higher risk of having underlying CAKUT.
Pietropaolo et al. 2025
TAKE HOME MESSAGE: Knowledge of the various forms of kidney involvement during fUTI will help clinicians make a prompt diagnosis and initiate the appropriate treatment to minimize complications such as sepsis, kidney scarring, and long-term kidney damage
